# Diet and serum lipids: changes over socio-economic transition period in Lithuanian rural population

**DOI:** 10.1186/1471-2458-11-447

**Published:** 2011-06-08

**Authors:** Vitalija Ramazauskiene, Janina Petkeviciene, Jurate Klumbiene, Vilma Kriaucioniene, Edita Sakytė

**Affiliations:** 1 Lithuanian University of Health Sciences, Academy of Medicine, Faculty of Public Health, Lithuania

## Abstract

**Background:**

Since regaining of independence in 1990, Lithuania has been undergoing substantial political, economic, and social changes that affected the nutrition habits of population. Dietary changes might have impact on the trends of dietary related risk factors of chronic diseases. The aim of the study was to compare trends in diet and lipid profile of Lithuanian rural population aged 25-64 during two decades of transition period (1987-2007).

**Methods:**

Four cross-sectional surveys were conducted within the framework of the Countrywide Integrated Noncommunicable Diseases Intervention Programme in five regions of Lithuania in 1987, 1993, 1999, and 2007. For each survey, a stratified independent random sample was drawn from the lists of the inhabitants aged 25-64 years registered at the primary health care centres. Altogether 3127 men and 3857 women participated in the surveys. 24-hour recall was used for evaluation of dietary habits. Serum lipids were determined using enzymatic methods. Predicted changes of serum cholesterol were calculated by Keys equation.

**Results:**

The percentage of energy from saturated fatty acids has decreased from 18.0 to 15.1 among men and from 17.6 to 14.8 among women over the period of 20 years. The average share of polyunsaturated fatty acids in total energy intake increased from 5.3% to 7.1% among men and from 4.9% to 7.3% among women. The mean intake of cholesterol declined among women. Favourable trends in fatty acids composition were caused by increased use of vegetable oil for cooking and replacement of butter spread with margarine. Since 1987, the mean value of total cholesterol has decreased by 0.6 mmol/l. Total dietary effect accounts for a 0.26 mmol/l (43.3%) decline in serum cholesterol among men and 0.31 mmol/l (50.8%) decline among women.

**Conclusions:**

Improvement in the quality of fat intake was observed in Lithuanian rural population over two decades of transition period. Positive changes in diet, mainly reduction in saturated fatty acids intake, contributed to decline in serum cholesterol level. Strengthening of favourable trends in nutrition habits in Lithuanian population should be one of the most important strategies of cardiovascular diseases prevention.

## Background

Cardiovascular diseases (CVD) are the leading cause of death in Lithuania [1]. In 2007, age standardized CVD mortality rate was 154.8 per 100,000 Lithuanian population aged 0-64 years. It was 3-fold higher than European average rate (48.7 per 100,000 population of the same age) [2]. Certain biological and behavioural factors are associated with risk of CVD. Epidemiological research has clearly demonstrated that high total cholesterol (TC), high low-density lipoprotein cholesterol (LDL-C), and low high-density lipoprotein cholesterol (HDL-C) are risk factors for developing CVD [3,4]. In Lithuania, the follow-up of cohort of Kaunas population aged 35-64 had demonstrated that hypercholesterolemia (HC) substantially contributed to the increase of CVD death risk in both genders [5]. Nutrition plays a major role in the development of dyslipidemias. Saturated fatty acids (SFA) and dietary cholesterol intake has been linked to an increased level of serum TC and LDL-C [6,7]. The replacement of saturated fat with polyunsaturated fatty acids (PUFA) has beneficial effect on lipid levels [8,9].

Since regaining of independence in 1990, Lithuania has been undergoing substantial political, economic, and social changes. These changes have influenced people's lifestyles and health [10]. The process of transition towards market economy and reduction in subsidies increased the prices of food products. Market globalization and aggressive advertising reduced the demand for local traditional foods. On the other hand, the availability of healthy foods such as vegetable oil, skimmed milk products, fruits and vegetables has increased. Changes in food market may have impact on the dietary intake of Lithuanians, particularly on quality of fat. Dietary changes may affect the lipid profile of Lithuanian population. Monitoring time trends of dietary habits and lipids level is very important for testing such a hypothesis. Lithuanian Countrywide Integrated Noncommunicable Diseases Intervention (CINDI) Programme offers such opportunity, as four regular cross-sectional surveys on representative population samples were carried out in the same rural regions of Lithuania between 1987 and 2007 [11].

The aim of the study was to compare trends in the diet and lipid profile of Lithuanian rural population aged 25-64 over two decades of transition period (1987-2007).

## Methods

### Study design and sample

This study used data of cross-sectional Lithuanian CINDI surveys carried out in five rural municipalities (Kaišiadorys, Kretinga, Kupiškis, Joniškis, and Varėna), randomly selected from the main administrative areas of Lithuania. The typical rural municipality consists of small town and surrounding villages with population ranging from 20 to 45 thousands. The surveys were conducted in 1987, 1993, 1999, and 2007. For each survey, a stratified independent random sample was drawn from the lists of the inhabitants aged 25-64 years registered at the primary health care centres of the region. In Lithuania, the majority of population is registered with a primary health care institution [1]. The samples were stratified by gender and 10-year age groups. Overall response rates ranged from 67.4% to 58%. Distribution of participants by survey year, gender and age are presented in table 1.

**Table 1:** Proportion of participants according to study year, gender and age.

	The survey year							
	
	1987		1993		1999		2007	
	
Age groups	Men (n = 976)	Women (n = 1085)	Men (n = 646)	Women (n = 821)	Men (n = 784)	Women (n = 975)	Men (n = 721)	Women (n = 976)
**25-34**	19.9	22.0	19.8	15.6	14.5	14.5	13.9	17.4

**35-44**	23.8	26.0	19.4	24.6	22.6	24.0	26.4	25.4

**45-54**	29.4	27.9	24.3	30.0	28.8	27.2	28.0	26.4

**55-64**	26.9	24.1	36.5	29.8	34.1	34.3	31.7	31.8

Survey methods strictly followed the WHO CINDI protocol [12]. Health examinations included interviews, clinical examinations, and laboratory tests.

The Lithuanian Bioethics Committee approved all surveys.

### Dietary assessment

A 24-hour dietary recall was used for the assessment of dietary intake. The data was collected by trained dietary interviewers. The participants were asked to recall what they had eaten during the previous 24-hours. The interviews in each survey were conducted on all days of a week, except Sundays. Food models, a validated picture book and household measures were used to quantify food portions sizes. The surveys were carried out during a whole year, so the seasons were equally represented. Nutrient values of food were calculated using the Lithuanian Food Composition Tables [13,14]. Under-reporters were defined by Goldberg cut-off for energy intake [15]. Persons with energy intake less than calculated basal metabolic rate were excluded from analysis.

In addition, some questions about nutrition habits were also included into the questionnaire. Information about fat used for cooking was elicited with the following question: 'What kind of fat do you mostly use for food preparation?' with response options: mostly vegetable oil, mostly margarine, mostly butter, mostly lard, I do not use any fat. The participants were also asked what kind of fat they mostly spread on bread. Possible responses were: I do not use any fat, I use margarine, spreads based on butter, butter, lard.

### Laboratory analyses

A venous blood specimen was taken to determine TC, LDL-C, HDL-C, and triglyceride (TG) levels. Subjects were asked to abstain from food intake at least 12 hours before the examination. In each survey the blood samples were taken in the morning. The serum TC was analyzed by enzymatic (CHOD - PAP) method [16]. The same method was used for analysis of HDL-C by precipitation of very low and low-density lipoproteins with the phosphowolfram acid and magnesium chloride mixture. Serum TG was analyzed by enzymatic (GOD-PAP) method [17]. LDL-C was calculated by Friedewald WT formula: LDL-C = TC - HDL-C - (TG × 0.45) [18].

All laboratory analyses were made in the same certified laboratory. Quality control measures were followed for estimation of lipids.

HC was defined when TC was 5.0 mmol/l or higher. The participants were asked when their blood cholesterol had been measured. The awareness about elevated blood cholesterol was assessed asking the following question: "Have you ever been told by physician that your cholesterol level is elevated?" The participants were considered to be treated if they reported use of lipid lowering medication. Those questions were asked in all surveys, except the first one.

### Statistical analysis

Statistical analyses were performed using the statistical software package SPSS 19.0 for Windows. Analysis was performed separately for men and women. Dietary variables included in the analysis were as follows: total energy intake (kJ/day), percent of total energy intake from protein, carbohydrates, total fat, fatty acids, as well as dietary cholesterol intake (in total grams and grams per MJ). The data were weighted to match the age distribution of the Lithuanian population aged 25-64 in 2007. The continuous variables are presented as means and standard deviations (SD) and categorical variables are expressed as proportions. The differences in age-adjusted means of dietary variables and lipids level between the surveys were assessed using analysis of variance and Bonferroni multiple comparison tests. A χ^2 ^test was used for testing the frequencies.

The Keys equation was used to predict the changes of TC over the study period:

SFA and PUFA are percentages of total calories provided by SFA and PUFA in the diet and Z is square root of dietary cholesterol (mg)/1000 kcal [19]. Δ means a difference in values of dietary variables between two surveys (1993 and 1987, 1999 and 1987, 2007 and 1987). The result of the equation was divided by 38.664 to convert predicted TC level from mg/dl to mmol/l.

## Results

From 1987 to 2007 both genders showed the reduction of total energy intake (by 9.7% in men and 14.0% in women) (Table 2). The percentage of energy (E %) from total fat remained stable over the study period. The most important nutrient variation concerned the energy intake from SFA, which decreased from 18.0 (5.1) E% to 15.1 (4.8) E% among men and from 17.6 (5.2) E% to 14.8 (4.9) E% among women. At the same time the average share of PUFA in total energy intake increased from 5.3 (2.3) E% to 7.1 (3.3) E% among men and from 4.9 (2.4) E% to 7.3 (4.0) E% among women. The mean intake of cholesterol declined in both genders. After adjustment for energy (mg/MJ) the significant difference was found only among women.

**Table 2:** Mean (SD) daily intake of energy, macronutrients, and cholesterol by study year and gender.

Energy, nutrients	Men				Women			
	
	1987	1993	1999	2007	1987	1993	1999	2007
**Energy (kJ)**	11615.6* (4249.7)	10149.4 (3637.5)	11069.6* (3776.2)	10493.1 (3416.6)	8860.2* (3240.1)	8236.6* (3078.6)	7835.9 (2815.2)	7618.4 (2632.9)

**Protein (E%)**	13.6* (3.7)	13.3* (4.1)	13.6* (4.5)	14.7 (4.1)	13.4* (3.5)	13.1* (3.6)	13.6* (4.2)	14.1 (4.2)

**Carbohydrates (E%)**	40.2* (11.1)	42.2*^#^ (11.3)	40.5* (10.9)	38.9 (11.5)	43.6 (10.7)	46.1* (11.1)	44.7* (10.9)	43.2 (11.9)

**Total fat (E%)**	45.2 (11.2)	43.3 (10.8)	43.8 (10.9)	44.8 (11.7)	42.8^#^ (10.4)	40.4 (10.5)	41.1 (10.4)	42.3 (11.3)

**SFA (E%)**	18.0* (5.1)	17.6* (5.0)	15.4 (4.8)	15.1 (4.8)	17.6*^#^ (5.2)	17.2*^#^ (5.3)	14.7 (5.1)	14.8 (4.9)

**MUFA (E%)**	17.8 (5.4)	16.8 (5.2)	16.5* (5.6)	17.3 (6.0)	16.2^#^ (4.9)	15.0* (4.9)	14.5* (5.2)	15.7 (5.6)

**PUFA (E%)**	5.3*^#^ (2.3)	4.7* (2.0)	7.0 (3.2)	7.1 (3.3)	4.9*^#^ (2.4)	4.2*^#^ (2.0)	7.3 (3.7)	7.3 (4.0)

**Dietary cholesterol (mg)**	456.9* (291.3)	432.9 (254.9)	436.1 (250.2)	423.1 (240.2)	360.2*^#^ (231.9)	361.4*^#^ (253.4)	289.3 (181.2)	276.4 (163.3)

**Dietary cholesterol (mg/MJ)**	39.6 (21.7)	44.6*^#^ (27.7)	39,6 (19,9)	40.0 (18.2)	41.1*^#^ (23.7)	43.8*^#^ (23.9)	37.1 (20.1)	36.8 (20.1)

*p < 0.05, compared to 2007; ^# ^p < 0.05, compared to 1999, using analysis of variance and Bonferroni multiple comparison tests;

Estimates are age-adjusted to the 2007 Lithuanian population

Abbreviations: SD - standard deviation; E%- percentage of total energy intake; SFA - saturated fatty acids; MUFA - monounsaturated fatty acids; PUFA - polyunsaturated fatty acids

**Figure 1:**
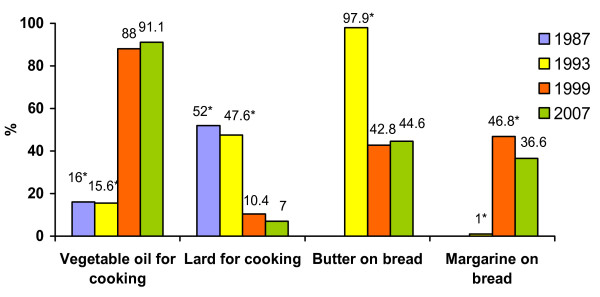
**The proportion of persons using different fats for cooking and on bread in 1987 - 2007**. *p < 0.001, compared with 2007.

The use of vegetable oil considerably increased over twenty years (Figure 1). The largest increase was observed between 1993 and 1999 (from 15.6% to 88.0% respectively). The use of lard for cooking changed in opposite direction. The proportion of people using mostly lard for cooking declined from 52.0% in 1987 to 7.0% in 2007. Butter was most common spread on bread in 1993. The proportion of persons spreading butter on bread halved in the next surveys because people replaced it by margarine.

The age-adjusted mean serum lipid levels of men and women are presented for each survey in the table 3. Over twenty years mean TC values has significantly decreased from 6.20 mmol/l (1.20) to 5.60 (1.16) mmol/l among men and from 6.12 (1.24) mmol/l to 5.51 (1.14) mmol/l among women. In both genders, the most significant reduction of TC occurred between 1993 and 1999. The changes in mean values of LDL-C were similar to the trends of TC. Mean value of HDL-C was significantly lower in 2007 compared to 1993 and 1999. The significant increase in mean TG was observed in both genders from 1987 to 2007.

**Table 3:** Mean (SD) serum lipid levels by study year and gender.

Serum lipids	Men				Women			
	
	1987	1993	1999	2007	1987	1993	1999	2007
**TC (mmol/l)**	6.20* (1.20)	6.05* (1.20)	5.51 (1.27)	5.60 (1.16)	6.12* (1.24)	6.03* (1.24)	5.56 (1.39)	5.51 (1.14)

**LDL-C(mmol/l)**	4.25* ^a^ (1.08)	3.94* (1.03)	3.37 (1.22)	3.57 (1.03)	4.18* ^a^ (1.13)	3.99* (1.12)	3.37 (1.36)	3.39 (1.00)

**HDL-C(mmol/l)**	1.33^a^ (0.46)	1.46 (0.43)	1.43 (0.46)	1.30^#^ (0.43)	1.42 ^a^ (0.46)	1.51 (0.47)	1.56 (0.41)	1.45^#^ (0.37)

**TG (mmol/l)**	1.41* (0.89)	1.40* (0.95)	1.60 (0.97)	1.63 (0.87)	1.15* ^a^ (0.60)	1.35* (0.75)	1.44 (0.77)	1.45 (0.60)

*p < 0.001 compared to 1999 and 2007; ^# ^p < 0.001 compared to 1993 and 1999; ^a ^p < 0.001 compared to 1993 using analysis of variance and Bonferroni multiple comparison tests

Estimates are age-adjusted to the 2007 Lithuanian population; SD - standard deviation;

Abbreviations: TC - total cholesterol; LDL-C - low-density lipoprotein cholesterol; HDL-C - high-density lipoprotein cholesterol; TG - triglycerides.

The contribution of fatty acids and dietary cholesterol on the change of serum TC was assessed using the Keys equation (Table 4). TC levels decreased by 0.60 mmol/l in men and by 0.61 mmol/l in women between 1987 and 2007. The changes in intake of SFA, PUFA and dietary cholesterol accounted for a 0.26 mmol/l (43.3%) decline in TC among men and for a 0.31 mmol/l (50.8%) among women. Intake of SFA explained 33.3% of TC decrease in men and 32.8% in women. The PUFA contributed to 10.0% decline of TC in men and 13.1% in women. Dietary cholesterol accounted for only 4.9% of the change in women and had no influence in men.

**Table 4:** Observed and estimated by Keys equation serum cholesterol (mmol/l) changes by study year and gender.

Year	Men					Women				
	
	Observed change	Estimated change	Contribution of changes			Observed change	Estimated change	Contribution of changes		
						
			Dietary cholesterol	SFA	PUFA			Dietary cholesterol	SFA	PUFA
**1987**	0	0	0	0	0	0	0	0	0	0

**1993**	-0.15	0.01	0.02	-0.03	0.02	-0.09	0.01	0.02	-0.03	0.02

**1999**	-0.69	-0.24	0	-0.18	-0.06	-0.56	-0.31	-0.03	-0.20	-0.08

**2007**	-0.60	-0.26	0	-0.20	-0.06	-0.61	-0.31	-0.03	-0.20	-0.08

The users of lipid lowering medication were excluded

Abbreviations: SFA - saturated fatty acids; PUFA - polyunsaturated fatty acids

In 1993, the prevalence of HC among men and women was significantly higher than in 1999 and 2007 (Table 5). In the last survey, one fifth of men and 30.5% of women with HC had their cholesterol measured within the last 12 months. In 1993, this proportion was extremely low (4.3% in men and 7.7% in women). The awareness about elevated TC has also increased in the period of 1993-2007 (from 0.4% to 10.5% in men and from 0.9% to 17.9% in women). The proportion of persons taking lipid lowering medication remained very low in 2007 (3.8% in men and 5.4% in women).

**Table 5:** Prevalence, detection, awareness and treatment of hypercholesterolemia in 1993-2007

Outcome measures	Men			Women		
	
	1993	1999	2007	1993	1999	2007
**Prevalence of HC (TC ≥ 5.0 mmol/l)**	79.1*^#^	62.6*	68.6	79.8*^#^	62.5	66.4

**Proportion of persons with HC having cholesterol measured during the last 12 months**	4.3*^#^	8.8*	20.5	7.7*	8.5*	30.3

**Proportion of persons with HC who were aware of their condition**	0.4*^#^	3.6*	10.5	0.9*^#^	6.4*	17.9

**Proportion of persons with HC who were taking lipid lowering medication**	0.4*	0.9*	3.8	0.5*	1.2*	5.4

*p < 0.05, compared to 2007; ^#^p < 0.05, compared to 1999

Abbreviations: HC - hypercholesterolemia; TC - total cholesterol

## Discussion

This study, based on four cross-sectional surveys, carried out during the past 20 years in Lithuania, indicated improvement in the quality of fat intake. This improvement was associated with significant changes in the consumption of fat containing foods. The changes in fat quality had serum cholesterol lowering effect at population level.

At Soviet time, consumption of fat milk and meat products was very high, while intake of vegetables and fruits was low in Lithuania [20]. The fatty acid pattern of the diet was unfavourable due to high share of SFA in total energy intake (18 E%). The most remarkable changes in nutrition habits of Lithuanians occurred in the first decade of transition period (1990-1999). In former Soviet Union lard was traditionally used in cooking due to shortage of other kinds of fat on the market. The great increase in availability of vegetable oil and affordable prices resulted in increased consumption of vegetable oil instead of lard [21,22]. Changes in the type of bread spreads were also substantial. At Soviet time, the majority of Lithuanians spread butter on bread because no soft margarine was available on the market and the price of butter was relatively low. In the early 1990s, various kinds of margarine became available in grocery stores. The price of butter increased and was considerably higher compared to margarine. This resulted in the shift from butter to margarine. In the last years, the supply of butter-based spreads increased in the Lithuanian market and consumption of margarine started to decline again [22]. The decrease in popularity of margarine could be partly explained by impact of media advertising butter as an ecological food product and introducing margarine as an unhealthy food product with a lot of additives.

The favourable changes in food habits contributed to the positive trends in fat intake of Lithuanian rural population. The proportion of PUFA in daily energy intake increased while the proportion of SFA as well as in dietary cholesterol intake decreased. During 20-year period, total fat intake almost did not change. We had no possibilities to analyze the trends in other important sources of fat such as meat and cheese because the questions about consumption of those products were incomparable between the surveys. However, the data from Lithuanian health behaviour monitoring indicate the increase in consumption of meat and cheese in population [22,23].

Similar trends in diet of populations have been also reported in other countries. Nutrition monitoring indicated a definite improvement in the diet of adult Finns, particularly in fatty acids composition. Over the last 25 years, the share of SFA in daily energy has decreased from 18.3% to 13% among men and from 17.6% to 12.1% among women. The intake of PUFA has increased from 4.3 E% to 5.9 E% among men and from 4.3% to 5.6% among women [24]. Because of health promotion, including promotion of healthy diet, the type of fat consumed has changed due to the increase in popularity of skimmed milk, low-fat margarines and low-fat cheeses [25]. Dietary surveys carried out in France demonstrated a significant improvement in fat quality of diets with an increase of the PUFA/SFA ratio, and a reduction in dietary cholesterol intake [26]. Reduction of animal fat consumption and an increase in vegetable fat intake were observed in Swedish, Polish, Czech, and in other European populations in the past decades [27-29].

Changes in diet have been in line with the observed decline in cholesterol levels. In Lithuania, the mean TC value has decreased by 0.60 mmol/l over the last 20 years. The contribution of dietary changes on the change of serum TC estimated by Keys equation was 43.3% in men and 50.8% in women In Finland, the 25-year decreasing trend in TC level and dietary impact on TC change were higher compared to our data [24]. The effect of dietary factors was estimated to account for 62% of reduction in TC among men and for 56% among women. The estimation of decline in TC was improved when the effect of trans-fatty acids was added into analysis [24]. The intake of trans-fatty acids was not considered in our study because Lithuanian Food Composition Tables do not have data on trans fatty acids content. There are other dietary factors affecting serum cholesterol level. The intake of plant sterols has been shown to have serum TC lowering effect [30]. Lithuanian health behaviour monitoring data showed the increasing trend in consumption of fresh vegetables and fruits in Lithuania [22,23].

The decrease in serum TC and in LDL-C among US adults between 1976-1980 and 1988-1994 was consistent with decrease in dietary intake of saturated fat and cholesterol [31]. Dietary data from NHANES 1999-2002 demonstrated only a small change in the intake of those nutrients [32]. The use of lipid lowering medication increased significantly in US between 1988-1994 and 1999-2006 [33]. In the last survey 38% of persons with high cholesterol reported medication use whereas 16% of respondents were under such treatment in 1988-1994. US researchers concluded that the decreases in TC and LDL-C over the last decade might have been influenced more by increased medication use rather than by dietary changes [32]. In Finland, the use of lipid lowering medication accounted for 16% of TC changes among men and 7% among women [24]. The use of statins was very low in Lithuania. In 2007, only 4.5% of respondents with HC reported the use of lipid lowering medication. Exclusion of persons under treatment from analysis did not influence the observed changes in the mean values of TC (Table 4).

The significant decrease in LDL-C was observed in Lithuanian rural population. This is in line with data from numerous studies, which have shown that replacement of SFA with PUFA leads to lower serum LDL-C [34]. According to our study, mean TG level has increased whereas mean HDL-C level has decreased in both genders since 1993. The increasing trends in prevalence of overweight and obesity in men and no changes of this indicator in women were found in Lithuanian adult population [22]. The evidence indicates that high TG levels and low HDL-C levels are associated with increased body weight [35]. Therefore, changes in body weight may partially account for the trends in TG and HDL-C levels in men. Similar trends in women could be to some extent explained by the increase in simple sugars intake. Lithuanian health behaviour monitoring data show the increase in consumption of confectionery among women over the last decade [22,23]. Evidence suggests that diets enriched in simple sugars create a metabolic state that is characterized by elevated TG and reduced HDL-C [36].

The findings of our study suggest that despite some positive changes in food habits the substantial part of population in Lithuanian rural regions does not meet the recommendations on healthy nutrition [37]. High intake of fat, especially saturated, as well as cholesterol is still the main problems of Lithuanian diet. Although TC and LDL-C levels showed the decreasing trends, they are too high and substantially contribute to increased CVD risk of Lithuanian population.

Healthy nutrition is among the most important challenges for public health in Lithuania. In 2003, the Government of the Republic of Lithuania approved the State Food and Nutrition Strategy and Action Plan for 2003-2010. The main goal of this Strategy was to protect health of the people and to reduce the prevalence of diseases related to unhealthy nutrition. The food based dietary guidelines for Lithuanian consumers were published to help people to choose food that meet recommendations. Those guidelines encourage limitation of consumption of fat, especially animal fat, meat products and promote vegetables and fruits consumption.

Our study has some limitations. The validity of 24-hour recall used for dietary survey may be affected by the memory and co-operation ability of respondents as well as by skills of interviewers [38]. In order to increase the accuracy of the data, food picture book and models were used to facilitate the estimation of portion sizes. The interviewers underwent special training course. As the surveys were conducted during all seasons of the year, the seasonal variations in dietary intake were considered. Under-reporting is one of the main limitations of 24-hour recall method [38]. However, under-reporting mainly affect crude nutrient intakes. In this study, the trends in nutrient intakes expressed as percent of energy were analysed. Our findings on nutrient intakes are consistent with the results of National Nutrition Surveys [39,40].

## Conclusions

The improvement in the quality of fat intake, in particular, reduction of saturated fatty acids and cholesterol intake and increase in polyunsaturated fatty acids intake has been observed in Lithuanian rural population over two decades of transition period. The positive changes in diet contributed to decline in serum cholesterol level. The strengthening of favourable trends in nutrition habits in Lithuanian population should be one of the most important strategies of CVD prevention.

## List of abbreviations

CINDI: Countrywide Integrated Noncommunicable Diseases Intervention; CVD: cardiovascular diseases; E%: the percentage of energy; HC: hypercholesterolemia; HDL-C: high-density lipoprotein cholesterol; LDL-C: low-density lipoprotein cholesterol; MUFA: monounsaturated fatty acid; PUFA: polyunsaturated fatty acid; SD: standard deviation; SFA: saturated fatty acid; TC: total cholesterol; TG: triglyceride

## Competing interests

The authors declare that they have no competing interests.

## Authors' contributions

JK and JP have made substantial contributions to conception and design of the manuscript. VR and VK have been involved in data collection and in drafting of the manuscript. ES has carried out statistical analysis. All authors read and approved the final version of manuscript.

## Pre-publication history

The pre-publication history for this paper can be accessed here:

http://www.biomedcentral.com/1471-2458/11/447/prepub
